# Novel computational models offer alternatives to animal testing for assessing eye irritation and corrosion potential of chemicals

**DOI:** 10.1016/j.ailsci.2021.100028

**Published:** 2021-12-05

**Authors:** Arthur C. Silva, Joyce V.V.B. Borba, Vinicius M. Alves, Steven U.S. Hall, Nicholas Furnham, Nicole Kleinstreuer, Eugene Muratov, Alexander Tropsha, Carolina Horta Andrade

**Affiliations:** a LabMol-Laboratory for Molecular Modeling and Drug Design, Faculdade de Farmácia, Universidade Federal de Goiás-UFG, Goiânia, GO, Brazil; b Laboratory for Molecular Modeling, UNC Eshelman School of Pharmacy, University of North Carolina, Chapel Hill, NC, USA; c Department of Infection Biology, London School of Hygiene and Tropical Medicine, London, WC1E 7HT, United Kingdom; d National Toxicology Program Interagency Center for the Evaluation of Alternative Toxicological Methods, NIEHS, Durham, North Carolina 27560, USA; e Department of Pharmaceutical Sciences, Federal University of Paraiba, Joao Pessoa, PB 58059, Brazil

## Abstract

Eye irritation and corrosion are fundamental considerations in developing chemicals to be used in or near the eye, from cleaning products to ophthalmic solutions. Unfortunately, animal testing is currently the standard method to identify compounds that cause eye irritation or corrosion. Yet, there is growing pressure on the part of regulatory agencies both in the USA and abroad to develop New Approach Methodologies (NAMs) that help reduce the need for animal testing and address unmet need to modernize safety evaluation of chemical hazards. In furthering the development and applications of computational NAMs in chemical safety assessment, in this study we have collected the largest expertly curated dataset of compounds tested for eye irritation and corrosion, and employed this data to build and validate binary and multi-classification Quantitative Structure-Activity Relationships (QSAR) models that can reliably assess eye irritation/corrosion potential of novel untested compounds. QSAR models were generated with Random Forest (RF) and Multi-Descriptor Read Across (MuDRA) machine learning (ML) methods, and validated using a 5-fold external cross-validation protocol. These models demonstrated high balanced accuracy (CCR of 0.68–0.88), sensitivity (SE of 0.61–0.84), positive predictive value (PPV of 0.65–0.90), specificity (SP of 0.56–0.91), and negative predictive value (NPV of 0.68–0.85). Overall, MuDRA models outperformed RF models and were applied to predict compounds’ irritation/corrosion potential from the Inactive Ingredient Database, which contains components present in FDA-approved drug products, and from the Cosmetic Ingredient Database, the European Commission source of information on cosmetic substances. All models built and validated in this study are publicly available at the STopTox web portal (https://stoptox.mml.unc.edu/). These models can be employed as reliable tools for identifying potential eye irritant/corrosive compounds

## Introduction

Chemicals employed in cosmetics, drugs, pesticides, household products, among others, need to be classified appropriately according to their potential ocular toxicity to ensure safety [1]. Eye irritation or corrosion are characterized by cell membrane lysis, coagulation, saponification, and chemical reactivity [2]. All of these characteristics are mediated by contacts between a chemical and the eye surface (cornea and conjunctiva) [3].

The Draize test, published more than 70 years ago [4], relies on *in vivo* exposure to rabbits’ eyes to classify chemicals according to their irritation/corrosion potential based on the damage caused within a well-defined timeframe [5]. However, this test relies upon qualitative scoring metrics of the severity and reversibility of highly subjective lesions, demonstrates poor reproducibility, and has questionable relevance to human exposure scenarios and human ocular biology [6]. Despite the scientific concern regarding the extrapolation of the observed results in rabbits to human eyes [7], the test is still used and recommended by the Organization for Economic Cooperation and Development (OECD).

The United Nations Globally Harmonized System (UN GHS) [8] proposes four categories to classify the chemicals: (*i*) Category 1 are compounds that cause irreversible eye effects within 21 days; (*ii*) Category 2A are compounds whose effects are reversible within 21 days; (*iii*) Category 2B are compounds whose effects are reversible within seven days; and (*iv*) No-Cat (NC) are compounds unable to cause eye corrosion or irritation.

Since the animal test ban in Europe for cosmetics ingredients in 2013, the development of alternative methods to substitute and reduce the number of animals in toxicological tests has become imperative [9]. The development of effective and efficient NAMs to animal testing [10] has been fueled in the last two decades by both public and political pressure [11] to employ the “Three Rs principles” to reduce, refine, and replace animal tests [12], and recent guidelines imposed by regulatory agencies create new demand for developing rapid, efficient alternative methods to animal testing [10]. Within this context, the 2018 ICCVAM strategic roadmap [13 ]called for the development of fit-for-purpose NAMs and the US EPA publicized its commitment to “eliminate all mammal study requests and funding by 2035” [14].

NAMs have been developed and made available for *in vitro* identification of ocular corrosives/severe irritants using alternative biological material including rabbit corneal cells (OECD Test Guideline 491) [15], isolated bovine corneas (OECD Test Guideline 437) [16], and a monolayer of Madin-Darby Canine Kidney (MDCK) cells (OECD Test Guideline 460) [17,18]. Other three-dimensional human tissue models such as the Reconstructed Human Cornea-like Epithelium (RhCE) test (OECD Test Guideline 492) and the Vitrigel-Eye Irritancy test (OECD Test Guideline 494) are approved for use in a bottom-up approach identifying substances not classified for ocular irritation. These tests provide varying coverage of the biology relevant to eye irritation and corrosion when compared to human ocular anatomy and physiology [19].

Computational models provide a fast and low-cost solution to obtain reliable predictions for the endpoint of concern when generated on high-quality curated data and properly validated [10]. A major computational approach, named Quantitative Structure-Activity Relationship (QSAR) modeling, employs various statistical and artificial intelligence (AI) approaches, such as machine learning (ML) and deep learning (DL) to generate models that can accurately predict the outcome of testing new compounds in a specific assay, based on their molecular features. In recent years, the growth in publicly available data enabled the development of highly robust and predictive models [20,21]. However, modeling toxicity is a complex task as the underlying mechanisms are not always clear [3,21]. For this reason, QSAR models are highly dependent on the quality and volume of the data [12] in the training set, proper chemical and biological curation of primary data is critical [22,23], and failure to follow these practices question the trustworthiness of models [6].

Recently, there have been many attempts to model eye irritation endpoints with varying degrees of success (see Table 1). Though many of the models showed good overall accuracy, most models were not compliant with the OECD’s guidelines for QSAR model development and validation [24], with models lacking the recommended use of an external set or Y-randomization [25-41], or not reporting the model applicability domain [28-41]. Many studies lack a rigorous curation and standardization of the chemicals used in the modeling, such as the study conducted by Verma et al. [25], resulting ultimately in unreliable predictions [42]. Additional problems include using unbalanced datasets, causing models to have an intrinsic bias toward the largest class [20,22]; and lack of model interpretation [20].^1^ These limitations make it impossible to fairly compare those tools with other peer reviewed and public QSAR models.

Our team has extensively worked on the development of QSAR models for toxicity endpoints and developed web applications to disseminate the use of these models, such as Pred-hERG [43] and Pred-Skin [44]. Considering the lack of reliable models for eye irritation and corrosion, herein, we have collected, curated, and integrated the largest publicly available eye irritation and corrosion datasets, used it to build predictive and rigorously validated ML and instance-learner models, integrated these models into a software package called STopTox (Systemic and Topical chemical Toxicity), and made it publicly available (https://stoptox.mml.unc.edu/). We offer these models as reliable computational tools developed under the NAMs paradigm for evaluating chemical hazard potential for eye irritation/corrosion.

## Materials and methods

### Dataset overview

**Fig. 1.**
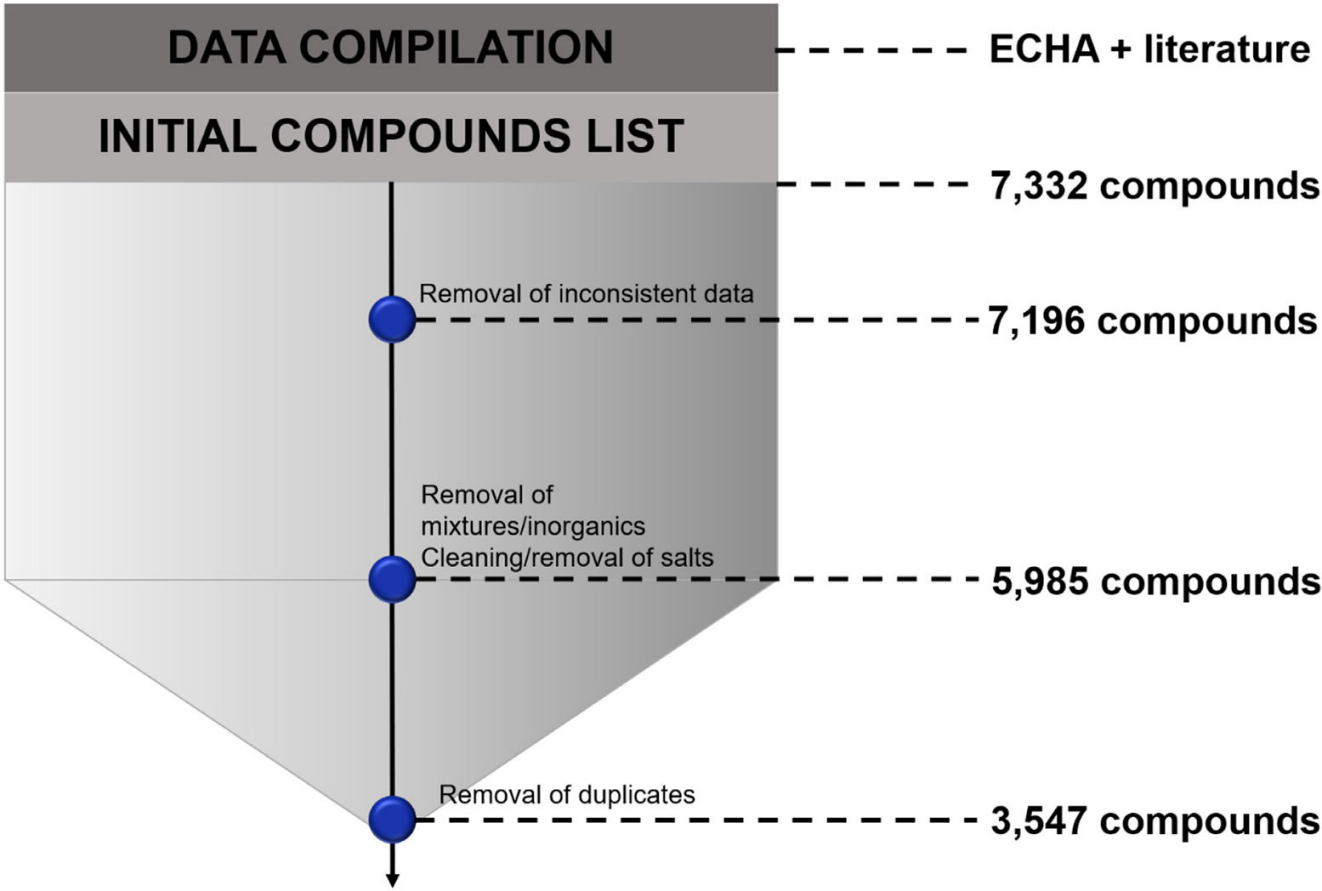
Data compilation and curation workflow.

The publicly available data from the European Chemical Agency (ECHA) (https://echa.europa.eu/) used in this work was graciously provided by Thomas Luetchtefeld [51]. Additional eye irritation-related data were also extracted from multiple literature sources [25,29,35,40,45,52-55], curated (see next section), and integrated. The ECHA dataset was initially composed of 18,428 records for 9,801 chemicals and we compiled 2,769 additional records from the literature. In the ECHA dataset, 5,238 records with imputed eye irritation/corrosion data from QSAR models, weight-of-evidence or read-across were excluded, leaving 7,332 records. Chemicals with inconsistent hazard classification data (*n* = 236) were removed. Inorganics (*n* = 330) and mixtures (*n* = 860), totalizing 1,190 entries, were also removed from the dataset. The data collected from the literature had high overlap with the ECHA data presenting 2,438 duplicates. No discordant duplicates in terms of hazard characterization were found between the ECHA data and the literature. These duplicates were carefully analyzed and only one entry per compound was kept. Furthermore, only studies following the OECD Test Guideline 405 [56] (*in vivo* data) were kept, with 3,547 records remaining after the data curation process (Fig. 1).

The final (unbalanced) dataset was composed of 3,547 compounds, of which 2,401 were classified as non-irritant/non-corrosive, 937 were classified as irritant (categories 2A and 2B) of which 209 were classified as corrosive (category 1). The GHS classification for irritant/corrosive compounds was only available for 1,248 compounds of the dataset, where 209 compounds were classified as category 1 members, 166 were classified as category 2A, and 84 as category 2B, whereas 789 were classified as NC. These compounds classified under GHS system were used to generate multiclass models.

Binary QSAR models using the unbalanced data typically lead to biased models. To overcome this, the negative class in the unbalanced dataset was under-sampled to balance the data set. We used the smaller group of irritant compounds as probes to search for the most structurally similar non-irritants selecting half of the irritant group (469 compounds). The remaining 468 compounds were randomly chosen from the rest of the initial non-irritant class to maximize the chemical space coverage. This similarity-based selection procedure was carried out in KNIME using Tanimoto coefficient in two stages: (i) generate a similarity matrix of chemical space between all the pairs of compounds; and then (ii) choose 469 non-irritants with the largest Tanimoto similarity to the nearest irritant and 468 via random selection. Such procedures allowed us to create the most challenging training set with structurally similar irritants and non-irritants to achieve the most rigorous model capable of separating these two classes from each other and including a fraction of more diverse non-irritants to provide broader chemical space coverage. The final dataset consisted of 1,874 compounds (937 irritants and 937 non-irritants). The same approach was performed to balance the data for the generation of QSAR models to predict eye corrosion, *i.e.*, the NC class of compounds was under-sampled using both structural similarity and random sampling, leading to a balanced data of 418 compounds (209 corrosive and 209 non-corrosive).

### Data curation

The compiled data was carefully curated and inspected according to protocols proposed by Fourches et al. [42,57]. Briefly, counter ions were stripped, mixtures and inorganics were removed, and specific chemotypes such as nitro groups and aromatic rings were standardized. Duplicates were identified, carefully analyzed, and only one entry was kept if biological responses were similar. The curation steps were implemented in the KNIME analytics platform (https://www.knime.com/) using in-house workflows. ISIDA Duplicates [58] was used to identify structural duplicates and ChemAxon Standardizer (v.16.5.16.0, ChemAxon, Budapest, Hungary, http://www.chemaxon.com) was used to standardize the chemical structures.

### Cluster analysis

A 50 × 50 neuron self-organizing map (SOM) was generated using the open-source software Data Warrior (http://www.openmolecules.org/) [59] and employing SkelSpheres descriptors (http://www.openmolecules.org/help/similarity.html) [60]. Data Warrior software was used to cluster compounds that were colored according to their Global Harmonization System (GHS) [8] class, in order to provide an overview of the chemical space.

### Molecular descriptors

We employed RDKit whole-molecule descriptors, Morgan, MACCS, and Dragon to develop QSAR and MuDRA models. SkelSpheres descriptors were calculated and used to cluster compounds in the SOM cluster analysis.

### SkelSpheres

Skeleton Spheres descriptors [60] were calculated through the Osiris Data Warrior software (http://www.openmolecules.org/). SkelSphere is a 1,024 bin byte-vector descriptor that, despite being time- and memory-consuming, is more suitable than the other descriptors to perceive fine similarities. It also considers stereoisomers and has fewer hash collisions due to its higher resolution. The SkelSphere descriptor was calculated prior to the SOM generation to better understand and cluster the compounds of the modeling dataset and to visualize GHS classification labels.

### RDKit molecular descriptors and fingerprints

In KNIME, a collection of 117 different RDKit molecular descriptors were calculated for the dataset followed by the removal of invariant descriptors and descriptors with a correlation higher than 0.9. MACCS structural keys [61] are implemented in the RDKit module available in the KNIME platform, as well as Morgan fingerprints [62]. RDKit provides 166 publicly available structural keys to represent molecules, and, for the Morgan fingerprint, it is possible to define the number of bits to encode the fingerprints as well as the radius, as the Morgan fingerprint is a circular fingerprint similar to ECFP and FCFP fingerprints family. For this study, Morgan fingerprints were generated using radius of 2 and 2048-bits length.

### Dragon descriptors

Version 5.5 of Dragon software (Talete SRL, Milan, Italy) was used to generate all the 0D, 1D, and 2D descriptors provided by the software, totaling 2489 descriptors [63]. After the descriptors were calculated, invariant descriptors and descriptors with a correlation higher than 0.9 were also removed prior to the model generation step.

### QSAR modeling

QSAR models for eye irritation and eye corrosion were generated employing a variety of chemical descriptors and algorithms. Binary and multiclass models were generated through the following steps: (i) data curation, preparation, and analysis; (ii) model generation and validation; (iii) model selection. To validate the method, we applied a 5-fold external cross-validation, where the curated dataset is divided into five equal-sized parts with an 80%/20% split between the modeling and test sets; this process is iteratively repeated until all parts of the dataset are used once as a test set. It is important to note that only the modeling set is used to generate the model; hence, during the 5-fold cross-validation procedure, compounds from the test set are not used in the generation of the models whatsoever and are solely reserved for the test set. Best models were carefully selected according to acceptable threshold values for all statistical metrics (for our purposes, this was set at 0.6). In addition, 10 rounds of Y-randomization were conducted to assess if the results were obtained by chance via annotating the statistical characteristics of the shuffled-labels models. Binary models were built for both corrosive and irritant classes of chemicals. Compounds classified as NC were used as non-corrosives and non-irritants as well.

### Algorithms

Both RF [64] and MuDRA [65] algorithms were applied. RF is a well-known ensemble decision tree learning algorithm, while MuDRA is an instance-based learning process. MuDRA does not build an underlying model to make its predictions but performs an instantaneous classification of known irritant/corrosive and non-irritant/non-corrosive compounds based on their similarity range and nearest neighborhood. An in-depth explanation of how the MuDRA method can be applied can be found elsewhere [65]. Both methods are implemented in the KNIME analytics platform; RF is a built-in node provided by different developers, while MuDRA is implemented through the integration between KNIME platform and Python scripting language via built-in nodes for this purpose.

### Statistical evaluation of models

The predictive power of both binary and multiclassification models was performed based on the output of the models during their respective validation processes. As described above, the 5-fold external cross-validation procedure was chosen to validate the models in this study. Hence, the statistical analysis is based on the collected results of predictions made in each fold of the cross-validation approach. For the multiclassification models, the same metrics were calculated, but considering the confusion matrix and comparing each class against all. The statistical metrics and the respective formulas are described below.


$$Se=\frac{TP}{TP+FN}$$

$$Sp=\frac{TN}{TN+FP}$$

$$CCR=\frac{Se+Sp}{2}$$

$$PPV=\frac{TP}{TP+FP}$$

$$NPV=\frac{TN}{TN+FN}$$

$$F_{1}=\frac{2TP}{2TP+FP+FN}$$

$$MCC=\frac{\left(TP\times TN)-(FP\times FN\right)}{\sqrt{\left(TP+FP)(TP+FN)(TN+FP)(TN+FN\right)}}$$

$$Coverage=\frac{Reliable\;predictions}{Total\;predictions}$$

Here, TP and TN are true positives and negatives, respectively; FP and FN are false positives and negatives, respectively. *Se* stands for the sensitivity of the models, which is the correct identification of positive samples, while *Sp* is the measure of the specificity of the models, evaluating the ability of the model to identify negative samples correctly. CCR stands for the correct classification rate, and are calculated as the arithmetic mean of *Se* and *Sp*. PPV means positive predicted value, while NPV means negative predicted value; these metrics evaluate the probability of certainty of a positive or negative prediction, respectively. F_1_ score, the harmonic mean of PPV and Se (aka precision and recall), evaluates the ability of the model to identify each instance correctly within the data. MCC encompasses the Mathews Correlation Coefficient and has been largely used as a goodness-of-fit in machine learning modeling tasks. MCC ranges from −1 to 1, being 0 equal to a random prediction.

The Coverage was calculated based on what we defined as reliable predictions, which means a prediction of a sample laying inside the applicability domain (AD) of the model, calculated through the formula below.

$$D_{T}=\bar{y}+Z\sigma$$

where D_T_ is a distance threshold, $\bar{y}$ is the average Euclidean distance of the k nearest neighbors of each compound of the training set, *σ* represents the standard deviation of the Euclidean distances and *Z* is an arbitrary parameter to control the level of significance. We set the default value of 0.5 for *Z*.

### Multiclass modeling

To build multiclass models, three classes were considered based on GHS classification: corrosive, irritant (comprised by classes 2A and 2B), and NC. The binary statistical metrics described above were computed for each of the three classes and averaged to report overall performance for the multiclass models.

### Virtual screening of CosIng and inactive ingredients database

The best models were applied to virtually screen the Cosmetics & Ingredients Substances Database (CosIng) [66], a European database of information about cosmetics and their ingredients. After curation, 4,780 compounds from CosIng were screened using our best performing models to identify compounds with the potential to cause eye irritation/corrosion.

The FDA inactive ingredients database (IID) set of compounds is freely available at https://www.fda.gov/drugs/drug-approvals-and-databases/inactive-ingredients-database-download. We also retrieved the data and curated it following the protocol described above. We applied the best models reported in this study to predict the ocular toxicity potential of the final IID set, composed of 4,673 inactive ingredients for pharmaceutical products.

### Dissemination

All workflows used in this work are available in the supplementary material for those who want to build models to other endpoints as well as for instructions about how to implement MuDRA along with Python and KNIME set-ups.

## Results and discussion

### Cluster analysis

**Fig. 2.**
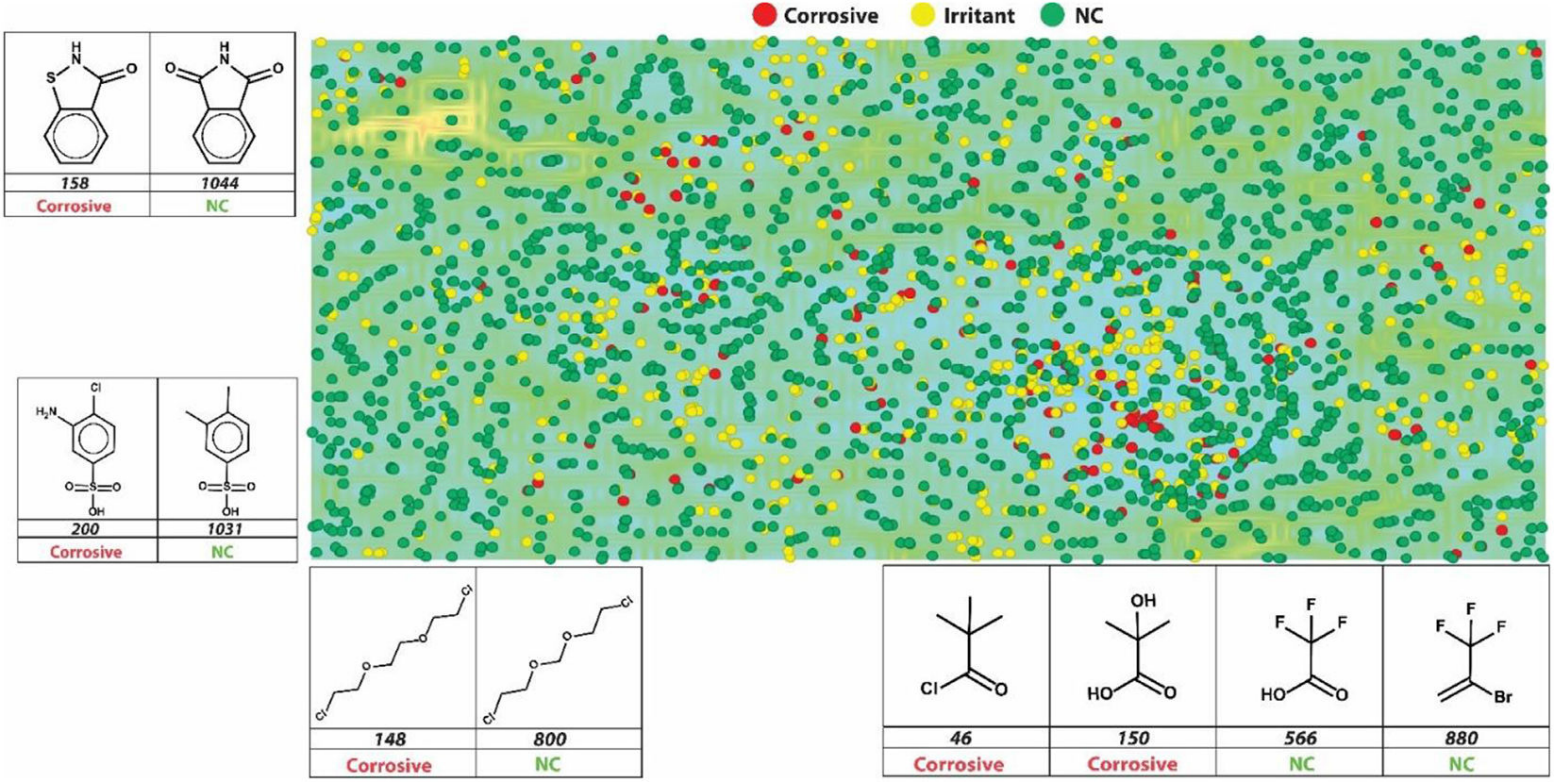
Graphical representation of a self-organized map for the chemical space covered by modeling set chemicals. Red circles represent corrosives, yellow circles represent irritants, and green circles represent NC class. Blue-green regions show compounds that share structural similarities compared to their neighbors, and yellow-orange-red regions represent an abrupt change in the chemical structure of the compounds compared to their neighbors. The dataset is notably complex; there are similar compounds belonging to different classes, which makes the construction of multiclassification models a challenge.

**Fig. 3.**
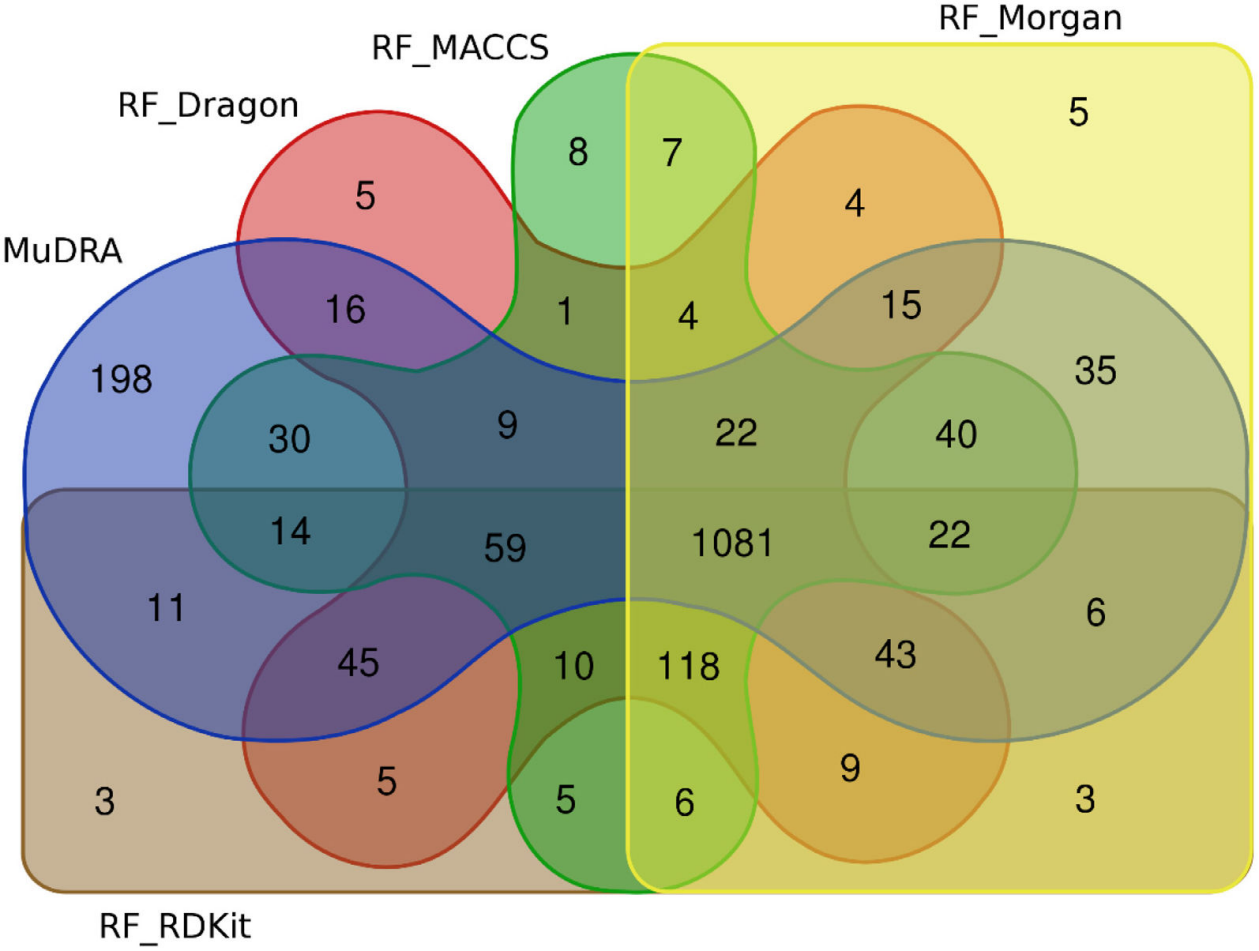
Venn diagram showing the overlap between correct predictions done by all models for the eye irritation dataset.

The SOM approach is an unsupervised classification technique that maps compounds to visualize their structural similarity. The structural map (Fig. 2) is colored by the three classes as defined by the GHS hazard classification system used to develop multiclass models. The highlighted compounds show that small structural differences can be observed in pairs of compounds belonging to distinct classes. Analyzing the background (shown in green in Fig. 2), the surrounding compounds are similar to each other (Tc = 0.85); major structural changes in the scaffold can also be observed (shown in yellow in Fig. 2), and structures with high dissimilarity are highlighted (shown in blue in Fig. 2). As seen in Fig. 2, the overall similarity between non-irritant and irritant compounds can be found across the whole map, sharing regions of the map in a non-compartmentalized way. This indicates that there are many activity cliffs in this dataset (as highlighted in Fig. 2). This type of dataset represents a challenge and although both RF and MuDRA are used to generate predictive models, they predict activity cliffs differently. As a prime example, 2,3-dihydro-1,2-benzothiazol-3-one (158 in Fig. 2) and 2,3-dihydro-1H-isoindole-1,3-dione (1044 in Fig. 2) are respectively within corrosive and NC categories, but share the same structural region in the SOM. Another example is 3-amino-4-chlorobenzene-1-sulfonic acid (200 in Fig. 2) and 3,4-dimethylbenzene-1-sulfonic acid (1031 in Fig. 2) pair, both in the same region of the SOM but respectively categorized as corrosive and NC. The same can be observed for the aliphatic compounds 1-chloro-2-[2-(2-*chloroethoxy*)*ethoxy*]ethane (148 in Fig. 2) and 1-chloro-2-[(2-*chloroethoxy*)*methoxy*]ethane (800 in Fig. 2), grouped together within the same region of the structural map and respectively classified as corrosive and NC. However, regions of chemical space are clearly enriched for particular categories, lending support to the application of QSAR modeling approaches while highlighting the necessity of nonlinear AI methods to identify the complex feature combinations that will discriminate categories.

### QSAR modeling

#### Binary models

In this study, we built five binary models for eye irritation and five binary models for eye corrosion. For each endpoint, we built RF models using four molecular descriptors described in the methods section as well as one MuDRA model. The AD of each model was calculated, with an exception for the MuDRA method as it is an instance-based modeling approach.

As one can see, models generated using the RF method for both endpoints presented similar metrics. However, they were outperformed by Models 5 and 10, generated using the MuDRA method, which was in agreement with the advanced performance of MuDRA in comparison with other QSAR methods as reported by Alves and colleagues previously [65]. The binary models for eye corrosion showed higher predictivity. This could be because the eye corrosion dataset is smaller as compared to the eye irritation dataset.

Obtaining high PPV values is crucial when dealing with toxicological endpoints as they indicate the ability of models to accurately predict toxic compounds. For eye irritation, we can see that PPV values ranged from 0.70 to 0.90 (with the lowest value from Model 2 and the highest value from Model 5). For eye corrosion, PPV values ranged from 0.65 to 0.88 (with the lowest value from Model 6 and the highest value from Model 9). Overall, PPV values were above acceptable thresholds and reached high values (0.9), meaning that a prediction made by the two best models generated in this study for both eye irritation and corrosion would be correct with more than 85% certainty.

Likewise, high NPV values are equally important as they provide the certainty of the prediction made by the model regarding the nonirritant/non-corrosive classes. Classifying a molecule correctly as nonirritant/non-corrosive is very important as an incorrect prediction could lead to eyes being damaged. NPV values for the models built in our study ranged from 0.77 to 0.85 for eye irritation and from 0.68 to 0.83 for eye corrosion. This shows that negative predictions made using our best models have at least 83% certainty.

In analyzing sensitivity and specificity, other studies have reported that specificity values are usually higher than sensitivity values [45,67]. Here the sensitivity values’ range was 0.77–0.89 for eye irritation and 0.61–0.84 for eye corrosion, while specificity values’ range was 0.56–0.86 for eye irritation and 0.71–0.91 for eye corrosion. In our study, all models for eye irritation showed sensitivity values higher than specificity. For the eye corrosion models, the same pattern was observed only for Model 7 and Model 8, otherwise specificity value was higher than the sensitivity value.

An additional cluster analysis was conducted to further investigate the better performance of MuDRA when compared to the models generated using RF for eye irritation and eye corrosion endpoints. In this analysis, it was noticed that all compounds belonging to the biggest cluster of compounds of eye irritation dataset (six irritants and 11 non-irritants) were correctly predicted by MuDRA. Meanwhile, from those 17 compounds within eye irritation dataset, the models built with RF algorithm combined with one type of molecular descriptor (Dragon, MACCS, Morgan, or RDKit) mis-predicted on average 7 of them (see Supplementary File 9).

**Fig. 4.**
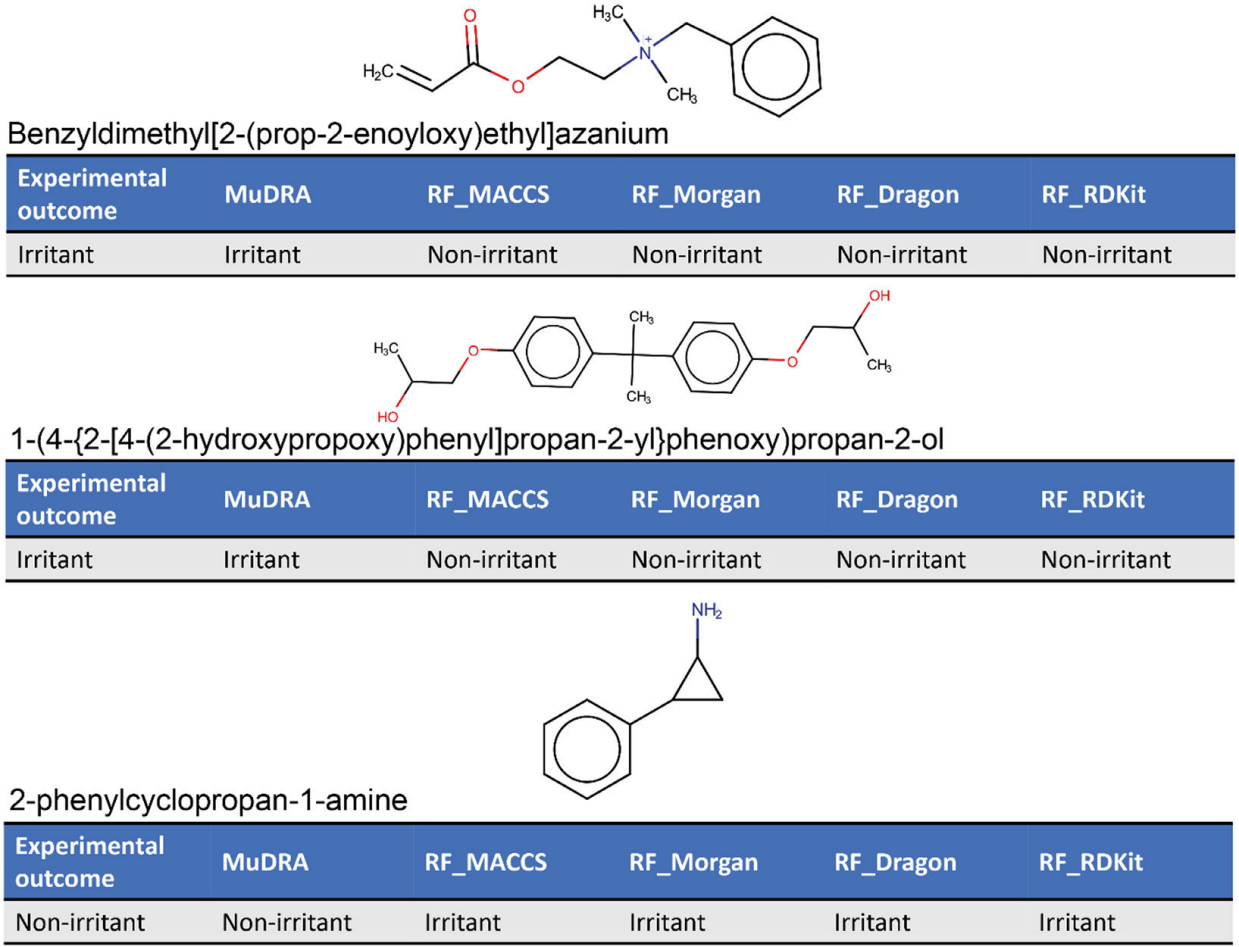
Example of compounds correctly predicted only by MuDRA.

Fig. 3 compares 1,646 correct predictions made by MuDRA, 1,446 correct predictions made by RF_Dragon (Model 3), 1,436 correct predictions made by RF_MACCS (Model 4), 1,420 correct predictions made by RF_Morgan (Model 2), and 1,440 correct predictions made by RF_RDKit (Model 1). It shows that MuDRA was able to correctly predict 198 compounds that the other models were not. It is important to note that 1,081 correct predictions were shared by all models. This reinforces the importance of data curation process as well as the use of best practices for QSAR modeling. On the other hand, when the overlap between all mis-predicted compounds was checked, it was noticed that 38 compounds (25 irritants and 13 non-irritants – see File S9) mis-predicted by all models were predicted correctly only by MuDRA (Fig. 4).

Moreover, we observed that MuDRA was more accurate than RF models when making predictions for certain chemical classes, such as long chain hydrocarbons and fatty acid derivatives (Fig. 4), such as ethyl tetradecanoate, 1,6-dioctyl-hexanedioate, 2-methylpropyl octadecenoate, and 2-[2-(*nonanoyloxy*)*ethoxy*]ethyl nonanoate. However, as this cluster was composed by only 17 compounds, this is not enough to assure MuDRA superiority over RF models. Overall, MuDRA uses a broader descriptor space, which is able to capture more rigorously the structural differences between compounds, to identify the nearest neighbor, read-across it, and then return a more accurate prediction.

#### Multiclass models

Using the data and the GHS labeling system, multiclassification models were generated. We used three classes based on the GHS classification: corrosive, irritant (comprised by classes 2A and 2B), and NC. Table 3 shows the overall statistical metrics for all multiclass models built in this study, averaged across the binary metrics for each class performance.

Model 15, generated using the MuDRA method, outperformed the other RF models in all statistical metrics except sensitivity. Thus, the majority of generated models using RF were above the acceptable threshold. It is important to note that all metrics shown in Table 2 were calculated using each class’s mean. The statistical characteristics showed that all models performed poorly when classifying compounds on GHS classes 2A and 2B This class has also been shown to have the lowest reproducibility when analyzing replicate animal tests, demonstrating the potential unreliability of classifications that are based solely on one result. However, the MuDRA method was able to handle the complexity of the data better by exploring the neighborhood of each compound and classifying them based on the nearest neighbor compound.

We have shown that MuDRA models were the best performing models in this work. As an external evaluation of model performance, we have predicted a set of 118 compounds extracted from Yamaguchi and colleagues’ study [68]. After dataset curation and preparation to remove compounds that were also present in our dataset, 107 compounds could be predicted. All 73 corrosive compounds and the 38 irritant compounds in the dataset were correctly classified, as well as the remaining 6 NC compounds. To further validate our approach, we made predictions of three compounds found in the literature and not included in our modeling set, that had been reported as capable of triggering moderate to serious issues in human eyes [69]. The compounds are glutaraldehyde [70], Paraquat [71,72], and glyphosate [73]. All three were correctly predicted as being irritants. This reinforces the predictive power of the MuDRA approach and its applicability to important toxicological endpoints such as eye irritation/corrosion.

#### Virtual screening

As a further application of our models, we have retrieved and carefully curated 4780 compounds from the Cosing database and predicted their effects on eye corrosion / irritation using the MuDRA models; complete details of the results are available in the supplementary material. In summary, our prediction identified 2003 compounds with the potential to cause eye irritation. We also predicted the effects on eye irritation and corrosion of the Inactive Ingredients Database (IID) containing 4673 inactive ingredients using MuDRA based model. The subset of compounds used in the ophthalmic route of administration had 181 entries consisting of 76 unique ingredients. Among them, 24 were predicted as potential eye irritants and 12 as corrosive, where most of these are reported as a component of formulations such as ointments, solutions, suspensions, and eye drop products. The list of compounds predicted by our models as eye irritants and corrosion is available in the Supplementary Materials of the paper.

## Conclusions

Eye irritation and corrosion are important toxicological endpoints for assessing chemical safety in humans and animals and respective tests are mandated by many regulatory agencies for the approval of a variety of products. The standard animal test for the evaluation of this endpoint is still the *in vivo* rabbit Draize test, a method developed decades ago and considered cruel, unreliable, and with questionable biological relevance to human exposure scenarios. Therefore, we aimed to develop predictive computational models using thoroughly curated data that could serve as NAMs for predicting eye irritation/corrosion potential of chemicals. Data curation is an extremely important factor in the development of robust and predictive ML models and a considerable amount of time was devoted to curating the ECHA dataset to ensure high quality training/test data and to optimize the predictive power of the models generated. All the curated data and developed models are available in KNIME workflows within the Supplementary Materials. These models presented high statistical characteristics. We have applied our models to predict a large publicly available cosmetics dataset (CosIng) as well as an Inactive Ingredient Dataset of chemicals commonly found in cosmetics and drugs. From CosIng database, 2003 compounds were predicted to cause damage to the eyes as corrosive/irritants; on the other hand, among 76 unique compounds from the Inactive Ingredients Dataset related to the ophthalmic route, 12 were predicted as corrosive, and 24 were predicted as irritants. The predictions for these chemicals are publicly available in the Supplementary Materials that accompanies this publication. Moreover, the models generated here are publicly available at the STopTox web portal (https://stoptox.mml.unc.edu/). These models can be employed as reliable alternatives to animal testing for identifying potential eye irritant/corrosive compounds.

## Supplementary Material

02

01

03

04

05

06

07

08

10

11

12

13

14

16

15

17

18

19

20

21

22

23

24

25

26

27

28

09

## Figures and Tables

**Table 1:** Previously published QSAR models of eye irritation.

Author	Curation	Cross- validation	Y-rand or external set	AD	Number of compounds	Metrics	AI/Discriminant method	Descriptor	Year	Model availability
Basant et al. [45]	Yes	Yes	Yes	Yes	107	Training: 77–94% Test set: 72–87%	CT, RT	Padel	2016	Unavailable
Verma et al. [25]	No	No	External set only	Yes	816 training 86 test	CCR = 72.3%	DT	Molecular weight, logP, melting point, aqueous solubility, lipid solubility	2015	Unavailable
Liew et al. [26]	Yes	Yes	External set only	Yes	2108 split in multiple categories	Training: CCR = 65–100% Test: CCR = 41–69%	SVM, kNN	Padel	2013	Publicly available [46]
Wang et al. [27]	Yes	Yes	External set only	Yes	6015 training 1504 test	CCR = 0.92–95%	ANN, kNN, NB, SVM	Atom pair, estate fingerprint, CDK fingerprints, Klekota–Roth fingerprint, MACCS fingerprint, Pubchem fingerprint and substructure fingerprint	2017	Unavailable
Jing Lu [47]	No	No	External set only	No	1845 training 496 test	CCR = 68%	Read Across	Codessa	2017	Unavailable
Geerts et al. [29]	No	No	No	No	80	CCR = 60–80%	Third-part software	None	2018	Unavailable
Bhhatarai et al. [30]	No	No	No	No	1644	CCR = 74–80%	Third-part software	None	2016	Unavailable
Luechtefeld et al. [31]	No	No	External set only	No	929	DT, kNN CCR = 73%–100%		Pubchem2d fingerprint	2016	Unavailable
Luechtefeld et al. [32]	No	Yes	External set only	No	15,760	CCR = 98%	Read Across	Pubchem2d fingerprint	2018	Unavailable
Verma et al. [48,49]	No	No	External set only	No	2928	Training: CCR = 85% Test: CCR = 83%	ANN	ADMET predictor	2015	Unavailable
Worth and Cronin [34]	No	Yes	No	No	119	CCR = 60–73%	LDA, CT, LR	Molecular weight	2003	Unavailable
Cruz-Monteagudo et al. [35]	No	LOO	No	No	46	Acc = 80.43%	LDA	LogP	2006	Unavailable
Solimeo et al. [36]	Yes	Yes	No	No	75	CCR = 82–92%	RF, kNN	Dragon, MOE	2012	Available by request*
Patlewicz et al. [50]	No	No	No	No	29	R [2]= 0.702	ANN	Logcmc, logP, molvol, mas $n-mas	2000	Unavailable
Sugai et al. [38]	No	LOO	No	No	138	Acc = 86.3%, Validation = 74%	ALS	Physico-chemical descriptors	1990	Unavailable
Cronin et al. [39]	No	No	No	No	53	R [2] = 0.80	LDA, LR	ClogP, kappa indices, molecular connectivity indices	1994	Unavailable
Barratt et al. [40]	No	No	No	No	46	N/A	PCA	ClogP, mol. vol., Dipole moment,	1995	Unavailable
Abraham et al. [41]	No	No	No	No	91	R^2^ = 0.94	LR	Liquid vapor pressure, mr, *π*^2^, Σ*α*, Σ*β*, liquid hexadecane partition	1998	Unavailable

CT = Classification Trees; RT = Regression Trees; SVM = support vector machines; kNN = *k* -Nearest Neighbor; ANN = Artificial Neural Networks; NB = Naïve Bayes; LDA = Linear Discrimination Analysis; LR = Linear Regression; RF = Random Forest; PCA = Principal Component Analysis; ALS = Adaptative Least Squares LOO = Leave One Out; CCR = Correct Classification Rate; Acc = Accuracy; N*/A* = not applicable; R^2^ = Correlation coefficient.

* Compounds must be sent to the authors to be predicted.

**Table 2:** Statistical characteristics of binary QSAR models for eye irritation and eye corrosion assessed by 5-fold cross-validation.

			Binary models for eye irritation generated with RF algorithm						
Model	Descriptor	CCR	Se	PPV	Sp	NPV	F1	MCC	Coverage
1	RDKit	0.76	0.77	0.76	0.76	0.77	0.77	0.53	1
	RDKit-AD	0.77	0.78	0.77	0.76	0.77	0.77	0.53	0.96
2	Morgan	0.77	0.84	0.73	0.69	0.81	0.78	0.53	1
	Morgan-AD	0.72	0.88	0.70	0.56	0.81	0.78	0.47	0.91
3	Dragon	0.77	0.78	0.77	0.77	0.78	0.78	0.55	1
	Dragon-AD	0.77	0.79	0.76	0.75	0.78	0.77	0.54	0.97
4	MACCS	0.77	0.80	0.75	0.73	0.79	0.77	0.53	1
	MACCS-AD	0.76	0.81	0.74	0.71	0.79	0.77	0.52	0,99
Binary model for eye irritation generated with MuDRA algorithm									
Model	Descriptor	CCR	Se	PPV	Sp	NPV	F1	MCC	Coverage
5	Multi	0.88	0.89	0.90	0.86	0.85	0.90	0.76	1
Binary models for eye corrosion generated with RF algorithm									
Model	Descriptor	CCR	Se	PPV	Sp	NPV	F1	MCC	Coverage
6	RDKit	0.70	0.70	0.71	0.71	0.70	0.70	0.41	1
	RDKit-AD	0.75	0.61	0.86	0.89	0.68	0.72	0.52	0.88
7	Morgan	0.68	0.76	0.65	0.59	0.71	0.70	0.36	1
	Morgan-AD	0.75	0.76	0.81	0.75	0.69	0.78	0.5	0.85
8	Dragon	0.72	0.73	0.72	0.71	0.73	0.72	0.44	1
	Dragon-AD	0.76	0.67	0.84	0.86	0.69	0.75	0.54	0.92
9	MACCS	0.76	0.73	0.78	0.79	0.74	0.75	0.52	1
	MACCS-AD	0.77	0.64	0.88	0.91	0.71	0.74	0.57	0.98
Binary model for eye corrosion generated with MuDRA algorithm									
Model	Descriptor	CCR	Se	PPV	Sp	NPV	F1	MCC	Coverage
10	Multi	0.85	0.84	0.86	0.86	0.83	0.85	0.69	1

**Table 3:** Statistical characteristics of multiclass QSAR models for eye irritation and eye corrosion.

		Multiclass models generated with RF modeling method							
Model	Descriptor	CCR	Se	PPV	Sp	NPV	F1	MCC	Coverage
11	RDKit	0.62	0.61	0.62	0.63	0.62	0.51	0.21	1
	RDKit-AD	0.60	0.60	0.60	0.60	0.61	0.52	0.22	1
12	Dragon	0.63	0.66	0.63	0.61	0.64	0.52	0.31	1
	Dragon-AD	0.60	0.61	0.61	0.58	0.58	0.52	0.31	1
13	MACCS	0.65	0.63	0.65	0.66	0.64	0.55	0.38	1
	MACCS-AD	0.64	0.67	0.64	0.62	0.64	0.56	0.39	1
14	Morgan	0.63	0.67	0.63	0.60	0.64	0.50	0.39	1
	Morgan-AD	0.60	0.71	0.62	0.49	0.60	0.51	0.39	1
				Multiclass model generated with MuDRA modeling method					
Model	Descriptor	CCR	Se	PPV	Sp	NPV	F1	MCC	Coverage
15	Multi	0.74	0.60	0.84	0.87	0.89	0.62	0.57	1

## Footnotes

1 It is important to notice that there are some commercially available models such as ADC/Percepta (https://www.acdlabs.com/products/percepta/index.php) and Case Ultra (http://www.multicase.com/case-ultra) and freely available software tools such as Toxtree (http://toxtree.sourceforge.net/) and QSAR toolbox that do not fully disclose their parameters, as well as datasets and statistics (https://www.oecd.org/chemicalsafety/risk-assessment/oecd-qsar-toolbox.htm#Guidance_Documents_and_Training_Materials_for_Using_the_Toolbox).
